# Advances of plant and biomass extracted zirconium nanoparticles in dental implant application

**DOI:** 10.1016/j.heliyon.2023.e15973

**Published:** 2023-05-06

**Authors:** Nayem Hossain, Md Hosne Mobarak, Amran Hossain, Fardin Khan, Juhi Jannat Mim, Mohammad Asaduzzaman Chowdhury

**Affiliations:** a Department of Mechanical Engineering, IUBAT-International University of Business Agriculture and Technology, Bangladesh; b Department of Mechanical Engineering, Dhaka University of Engineering and Technology (DUET), Gazipur, Gazipur, 1707, Bangladesh

**Keywords:** Zirconium, Dental implant, Nanoparticles, NPs, ZrO_2_, Nanotechnology, Osseointegration

## Abstract

Nanoparticles are minimal materials with unique physicochemical features that set them apart from bulk materials of the same composition. These properties make nanoparticles highly desirable for use in commercial and medical research. The primary intention for the development of nanotechnology is to achieve overarching social objectives like bettering our understanding of nature, boosting productivity, improving healthcare, and extending the bounds of sustainable development and human potential. Keeping this as a motivation, Zirconia nanoparticles are becoming the preferred nanostructure for modern biomedical applications. This nanotechnology is exceptionally versatile and has several potential uses in dental research. This review paper concentrated on the various benefits of zirconium nanoparticles in dentistry and how they provide superior strength and flexibility compared to their counterparts. Moreover, the popularity of zirconium nanoparticles is also growing as it has strong biocompatibility potency. Zirconium nanoparticles can be used to develop or address the major difficulty in dentistry. Therefore, this review paper aims to provide a summary of the fundamental research and applications of zirconium nanoparticles in dental implants.

## Abbreviation

NPs –: Nanoparticles
Zr: Zirconium
ZrO_2_ –: Zirconium Dioxide
Ti–: Titanium
Ag –: Silver
Cu –: Copper
ZnO –: Zinc Oxide

## 1 Introduction

Since the discovery of nanomaterials, a branch of science that studies objects with a diameter of less than or equal to 100 nm, and the tiny size of the nanoparticles that give them some distinctive qualities, it has opened up a ton of research investigations that span several disciplines, including chemistry, physics, medicine, and more [1]. Because of their high surface energy, huge surface area to volume ratio, and relatively tiny size compared to the bulk material, nanoparticles have demonstrated unique catalytic, thermal, optical, electrical, and biological applications used in various sectors [2].

Nanotechnology has many potential benefits and possible threats to human health, so it is at the forefront of the rapid development of healthcare products [3]. Mainly, Zirconium Nanotechnology shows long-term clinical success from the beginning of the trial period compared to its peers [4]. Because of their durability and exceptional strength, ZrNPs are frequently utilized in dentistry due to zirconia's superior chemical stability, bio-compatibility, adequate fracture resistance, and flexural strength [5]. Dental implants greatly depend on the power of NPs, which zirconium NPs ensure. Comparative to other nanoparticle materials, zirconium NPs offer greater adjustability [6]. Also, due to decreased porosity and increased thickness, zirconia nanoparticles improved the coating's abrasion behaviour [7]. Both histological and cytological studies have shown that the level of toxicity is moderately low [8]. Despite the range of examined materials and test methodologies, no local or systemic adverse reactions related to the substance (Zr) or cytotoxic effects directly related to the presence of zirconia ceramics or precursors were found [9].

Zirconia implants are becoming more common in clinical practice, but they are not yet a standard procedure because of insufficient mechanical and scientific investigations [10]. The claims that nano-ZrO_2_ additions may have antioxidant and anticarcinogenic properties seem encouraging, given that the issue of potential nanoparticle toxicity is now being researched [11]. Studies evaluating the effectiveness of this addition in the creation of nano-ZrO_2_ bone substitute materials have also been conducted [12]. Despite numerous drawbacks and potentially dangerous factors, research on this phenomenon is highly reliable.

## 2 Importance of nanoparticles in dental implants

In recent years, dental implants have shown high appreciation clinically by using nanoparticles. It needs a small area for a correspondingly larger volume for the area/volume ratio [13]. Nanosomic materials are mainly intermediate materials of microscopic as well as molecular levels. It has significant advantages for decreasing the ratio of surface area and volume. For instance, if the surface area of the sphere increases by factor r^2^, the volume factor increases by factor r^3^. Then the ratio of them decreases consistently. Therefore, nanomaterials generate significant outer surface area by maintaining volume accordingly [14].

Nanoparticles have the highest potential for anti-bacterial effects. It can tolerate high temperatures and can ensure hard coating on the surface. Some particles, like Calcium phosphate, are physical and chemical similarities with some tissues like Dentin and enamel [15]. It is used in oral diseases, dental prostheses, and implants [16].

**Fig. 1:**
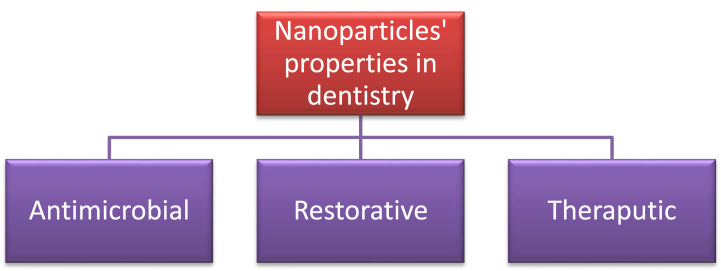
Nanoparticles' properties in dentistry [18].

Nanoparticles used in peri-implant tissue activate osteoclasts' activity. That means it helps to strengthen the tooth's tissues after getting a concussion. It regenerates the strength of dental movement [17]. Fig. 1 shows the properties of NPs in dentistry.

Nanoparticles give almost the same texture as natural teeth. Besides, it is pretty easy to make the fitted size as it originates for every possible shape [19]. It is also used in dental fillings such as root canals. Because it ensures a large surface area and presents antimicrobial, antiviral, and anti-fungal properties. Moreover, it enhances mechanical properties and strengthens bonds by improving toughness [20].

**Fig. 2:**
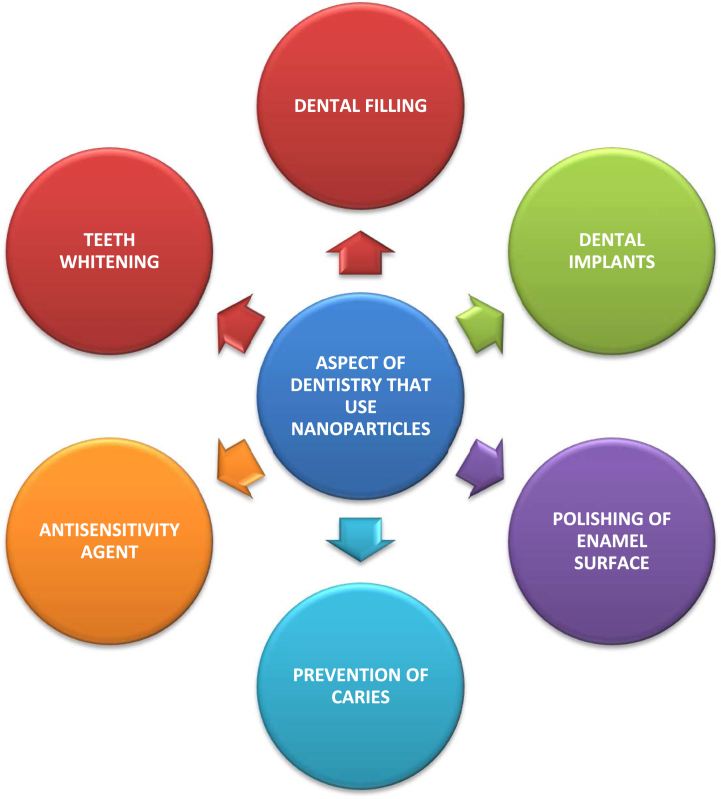
Uses in dentistry of Nanoparticles [19].

Fig. 2 shows the uses of nanoparticles in dentistry.

Some basic nanoparticles are used in Dental implants. They are.

### 2.1 Titanium-based nanoparticles

The capacity of a material to integrate with living tissue without having an unfavourable effect is known as biocompatibility, and it is one of the essential characteristics of any dental implant material [21]. As titanium-based nanoparticles have excellent biocompatibility, they are not likely to result in tissue rejection or an allergic reaction [22]. Titanium has good recognition of corrosion resistance and protection from distortion [23]. Due to their superior mechanical strength and remarkable osseointegration qualities, titanium dental implants have demonstrated exemplary performance. According to research, titanium-based nanoparticles can decrease inflammation in the surrounding tissue, lowering the chance of implant failure and speeding up healing [24]. Ti-based implants typically have their surfaces modified to prevent implant failures [25].

### 2.2 Silver-based nanoparticles

Silver nanoparticles have received a lot of attention in dentistry due to their unique features, such as antibacterial, antifungal, and antiviral qualities [26]. Dental implants are vulnerable to bacterial infections, which might result in implant failure [27]. The likelihood of conditions has been reported to be decreased by the use of silver nanoparticles in limiting bacterial development on implant surfaces [28]. So these particles give the same texture as the tooth but also help resist bacteria growth [29]. It also has caries inhibitory properties, which fight fibroblasts. Per day it releases a meagre amount of ions [30]. Silver nanoparticles have also been demonstrated to help the implant integrate with the surrounding bone tissue [31]. This is due to the ability of silver ions to promote bone cell development and differentiation, resulting in a more rapid and effective bone healing process around the implant [32]. Dental implants have also been coated with silver nanoparticles, increasing the implant's biocompatibility and resistance to corrosion and wear. Ultimately, the implant may work better over the long run [33].

### 2.3 Copper-based nanoparticles

One of its main advantages is the potential of copper-based nanoparticles in dental implants to stop bacterial infections [34]. Due to their susceptibility to bacterial infections, dental implants might experience problems like peri-implantitis and implant failure [35]. It has been demonstrated that copper-based nanoparticles can efficiently suppress bacterial growth on implant surfaces and stop the development of biofilms, which can lower the risk of infections [36]. Its toxicity level is also low, which is not alarming for oral health. The quantity of copper nanoparticle demand for dental implants is very, which does not create any problems [37]. It also has an anti-inflammatory element that has been used in medicine previously. It reduces inflammation, like redness, swelling, and pain [38]. The potential for copper-based nanoparticles to promote bone development and improve osseointegration of dental implants has also been investigated. Copper ions have been demonstrated to encourage bone cell differentiation and boost collagen formation, accelerating bone repair and implant integration [39].

### 2.4 Zinc based nanoparticles

Dental implantology, in particular, is becoming increasingly interested in zinc materials [40]. ZnO has very variation in sizes and shapes. For this reason, it helps to use different purposes. So it is straightforward to get adjustable sizes in ZnO NPs [41]. It has antibacterial activity for both Gram-positive and Gram-negative bacteria. Besides, it shows good activity with oral surfaces and textiles [42]. Antibacterial properties of zinc are known to help with infection prevention and healing after implant surgery [43]. Zinc can also promote bone formation, which is necessary for dental implants to last long [44]. Dental implants can also be coated with zinc-based nanoparticles to increase their tensile strength and resistance to abrasion [45]. Because of their biocompatibility and minimal toxicity, zinc-based nanoparticles are suitable for usage inside the human body [46]. They can be generated in large quantities at a reasonable cost because they are also relatively easy to synthesise [47].

### 2.5 Iron-based nanoparticles

Although Iron nanoparticles have not received as much research as other types of nanoparticles, they are also of interest in dental implantology. The formation of haemoglobin, which is vital for the transportation of oxygen in the blood, depends heavily on iron, an essential mineral [48]. It has corrosion protection behaviour which resists protecting teeth in the future. Besides, surface hardness and wear resistance characteristics give significance in dentistry [49]. Iron-based nanoparticles' capacity to stimulate bone development in dental implantology is one potential advantage. Osteoblasts, which are the cells responsible for bone formation, have been demonstrated to be encouraged by iron. This might facilitate better dental implants' long-term success by enhancing their integration with the surrounding bone tissue [50]. Magnetic characteristics of iron-based nanoparticles could be advantageous in creating novel dental implant materials. Targeted forces could be applied to magnetic dental implants to encourage the bone formation and speed up the healing process after implant surgery [51]. Iron-based nanoparticles are suitable for usage in the human body because they are biocompatible and have minimal toxicity, similar to their peers. To completely comprehend the possible advantages and hazards of employing iron-based nanoparticles in dental implantology and optimize their use in this context, further research is still required [52].

## 3 Synthesis approaches of zirconium nanoparticles from plants

A significant research focus throughout dental implant applications is the sustainable production of plant nanoparticles. Compared to traditional organic antibacterial agents, it is superior in terms of reliability, resilience, and heat resistance against bacterial pathogens apart from Staphylococcus aureus and *E. coli,* in addition to antifungal effectiveness against *C. albicans* and Aspergillus Niger [53].

The addition of organic material in the synthesis of ZrO_2_ NPs has drawn considerable attention due to its ecologically friendly, convenient, reliable, non-toxic, and cost-effective procedure, which provides a one-step approach for green synthesis methods. The organic biosynthesis and investigation of ZrO_2_ NPs is a significant aspect in investigating the effect of ZrO_2_ NPs on human beings and the environment, in addition to implying their utilization. Considering potential biocompatibility, materials have been studied for dental implants [54,55]. ZrO_2_ NPs are often synthesized by incorporating bottom-up and top-down approaches. Bottom-up or cooperative material synthesis fluctuates via atoms through the clusters to nanoparticles [56,57]. Sol-gel, spinning, vapour deposition (CVD), pyrolysis, and biosynthesis are the most common bottom-up techniques for synthesizing nanoparticles. The exceptionally high temperature and pressure parameters required for the synthesis process require considerable energy, which is another limitation of this approach. Nanoparticle stability, shape, and dimension may be precisely regulated by appropriately managing the temperature, pH, leaf extract concentration, metallic saturated salt concentration, and incubation length. The bottom-up method comprises the production of nanoparticles from tiny basic units, such as molecules and atoms, through the consciousness of atomic nuclei to existing nuclei, which integrate into nanosomic particles and utilize various chemical and biological processes. This category consists of a variety of methods also, including thermal oxidation, pyrolysis, radiation-induced, and nanotechnology [58]. This review mentions several plants that have been reported to be used in the production of zirconium nanoparticles. Table 1 shows the size distribution and synthesis approaches of plant extract medicated Zirconia NPs.

**Table 1:** Size distribution and synthesis of zirconia nanoparticles from several plant species [58].

Serial. No	Name of Plants	Part Used	Shape	Size (nm)	Reference
1	duchesneaindica	Leaves	spherical	80	59
2	*Camellia sinensis*	Leaves	spherical	25	60
3	Alliumcepa	Fruit	Baddeleyite	13.03–21.97	61
4	Lycopersiconesculentum	Fruit	Baddeleyite	20.48–21.37	62
5	Aloe Vera	Leaves	Spherical	50–100	63
6	*Curcuma longa*	Tuber	Chain	41–45	64
7	Lemon juice	Fruit	Quasi-spherical	20	65
8	chebulicmyrobalan	Seed	–	15.52	66
9	Pseudomonas aeruginosa	Leaves	spherical grains	6.41	67
10	*Helianthus annuus*	Seed	Monoclinic	40.59	68
11	Calotropisgigentea	Leaves	–	420	69

Despite the various chemical and physical approaches that can be implemented to create nanoparticles, each process has certain limitations. Nanotechnology and biotechnology are emerging to converge concerning nanoparticle photosynthesis. It has gained greater prominence because of the increasing demand for ecologically friendly material synthesis methods. The desire to achieve more excellent structure and size control for several uses has driven researchers to investigate this nanoparticle production method [69].

Creating nanoparticles using plant extract is non-toxic, rendering plants the best choice. Plant extracts like geranium, Aloe Vera, sun-dried Cinnamomum camphor, Azadirachtaindica, etc., help create silver and gold nanoparticles [70].

Due to the excessively straightforward, nontoxic, quick, ecologically safe, cost-effective, and simple one-step method to manufacture NPs, using plant material to synthesise zirconium dioxide nanoparticles generated great enthusiasm [71]. That biomolecule includes tannins, sugar, steroid, enzymes, phenols, amino acids, flavonoids, and sugar, typically obtained from natural extracts and for significant overall medical purposes. The plants also include diverse combinations of biomolecules that aid in the stability of ZrO_2_ nanoparticles [72].

**Fig. 3:**
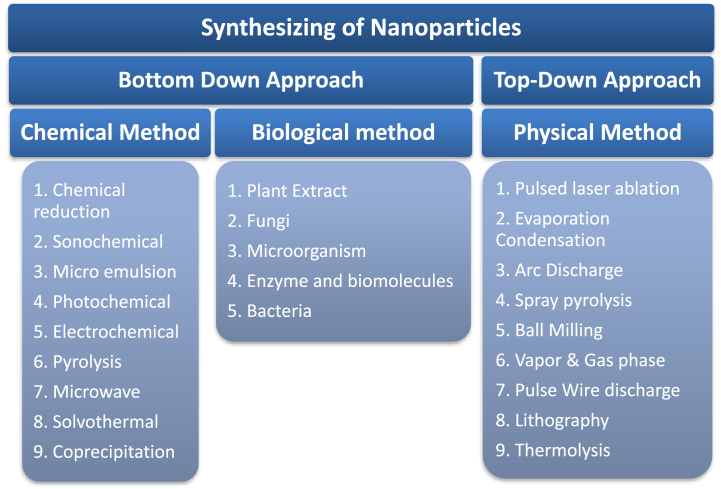
Synthesizing of nanoparticles [73].

Fig. 3 illustrates how several techniques have helped nanoparticles develop.

## 4 Characterization of synthesized ZrO_2_ nanoparticles

Although Zirconium nanoparticles have many advantages over traditional drug design, delivery, and medical diagnostics, nanomedicines present significant challenges for preclinical development. Nanoparticle constructs for medical applications are made up of a wide range of materials, and their small size, unique physicochemical properties, and biological activity frequently necessitate the modification of standard characterization techniques [74]. Researchers applied synthetic techniques to generate and describe ZrO_2_ nanoparticles, employing multiple approaches to recognize their size. The characterization of ZrO_2_ Nanoparticle synthesis uses specific critical characterization approaches. Such as X-ray diffraction (XRD), Surface morphological analysis, UV–visible analysis, scanning electron microscopy (SEM), transmission electron microscopy (TEM), antimicrobial analysis, thermal gravimetric analysis (TGA), and FTIR spectroscopic [[75], [76], [77], [78]].

### 4.1 Scanning electron microscopy (SEM) and transmission electron microscopy (TEM)

Scanning electron microscopy (SEM), images were used to calculate the volume of the produced ZrO_2_ nanoparticles. The most important terms for morphological characterization are scanning electron microscopy (SEM) and transmission electron microscopy (TEM). SEM and TEM are utilized for morphological characterization at the nanoscale to micrometre scale. SEM can provide electropositive information at the micron-scale and submicron-scale morphological characteristics [75]. A field-emission scanning electron microscope (FE-SEM) (Nova NanoSEM 230, Czech Republic) operating at 5.0 kV was used to evaluate the shape and size of the adsorbents [79].

### 4.2 FT-IR spectrum

Fourier transforms infrared spectroscopy (FTIR), which may be utilized to characterize the surfaces of the nanoparticles, can be used to determine the surface chemistry [75]. By analyzing their FT-IR spectrum using the SHIMADZU model in the diffused reflection form, researchers could analyze the manufactured samples' pattern of ZrO_2_ nanoparticles [76]. FTIR analysis was carried out using a spectrometer built in the USA by PerkinElmer in the frequency range of 500 cm^−1^ to 4000 cm^−1^ [77].

### 4.3 Atomic force microscopy (AFM)

Atomic force microscopy (AFM) can characterize nanoparticles at their atomic scale [75]. Green nanoparticles have been produced using AFM for dimension surface topography and granularity volume distribution [80]. This is a frequently used method for scanning probe microscopy. In the past, the experimental study of the interaction of nanoparticles with the AFM probe was fairly thorough, including measurements of the nanoparticles, modifications to the AFM tip, and nanoparticle manipulation [[81], [82], [83], [84]].

### 4.4 X-ray diffraction

XRD is used to ensure the crystal formation (crystal phases) and ordain the crystallization shape of each Phase. By employing the SSD160 1D Detector, the samples' XRD spectra were collected to determine the form of the produced ZrO_2_ nanoparticles [76]. Low (2q3) and high (2q of 10e90) diffraction angles were used in X-ray diffraction (BRUKER X-Ray) experiments with Cu-Ka radiation at 40 kV, 30 mA, and room temperature to detect the structural characteristics, purity, and oxide phases development of the catalysts [85].

### 4.5 Antimicrobial analysis

The Kirby-Bauer disk diffusion test technique, which adheres to ASTM E2149-01 standard, was employed to determine the synthetic nanoparticles' antibacterial characteristics. Bacterial cells were present in a 1000 CFU/ml sample, while the nanoparticle concentration was 200 mg/ml. To determine the suitability of the nanoparticles for dental implant applications, a test was conducted against gram-positive and gram-negative bacteria [77].

### 4.6 UV–visible analysis

A 200–1100 nm Shimadzu 1601 spectrophotometer. Energy-sensitive calculations and assertions for visible attributes, movement observations, absorption coefficient, and gap assurance [80]. On ZrO2 nanoparticles, optical absorption measurements were also carried out The wavelength range of 300–700 nm was used to determine the optical absorption coefficient. When compared to bulk ZrO2, it can be seen that the absorption edge is somewhat red-shifted, indicating that it is nearing visible area absorption. So, at higher wave-lengths, the samples are entirely transparent. A valence-to-conduction band transition causes an absorption peak in the UV spectra to appear at 320 nm [86].

### 4.7 Thermogravimetric analysis (TGA)

Thermal gradient analysis (TGA) is used to evaluate the thermal stability, thermal breakdown, and phase change of the nanoparticles [87]. The amount of mass lost by aerogel samples as a function of temperature is calculated using the thermogravimetric analysis (TGA), differential thermal analysis (DTA), and differential scanning calorimetry (DSC) methods. A temperature range of 1000 °C–1400 °C is usually used for thermal analysis [88,89].

### 4.8 Dynamic light scattering (DLS)

The size distribution and quality of nanoparticles and their surface charge are all characterized by DLS. The produced nanoparticles' polydispersity index may be studied and is also extremely helpful [87].

### 4.9 Energy dispersive spectroscopy (EDS)

The essential knowledge of the sample is provided by EDS, which may be utilized to assess the elemental composition of metal nanoparticles [87]. The proportion of elements in the sample was examined using EDS [90].

## 5 Importance of zirconium nanoparticles

The latest implantation technologies have improved. The characteristics that make titanium implants, such as cutting-edge medical technology, should keep their high success rates. In dental implantology, zirconia implants are increasingly employed instead of titanium implants. Zirconia is a suitable material for implants due to its low affinity for plaque, tooth-like colour, biocompatibility, and mechanical attributes [91].

Zirconium dioxide (ZrO_2_), also known as zirconia, is a material that has gained increasing attention in dentistry due to its unique properties and biocompatibility. Due to their excellent strength, hardness, biocompatibility, and transparency, zirconia nanoparticles are being researched for dental implants [92]. Several cell types, including fibroblasts and epithelial cells, have been reported to benefit from zirconium nanoparticles in terms of increased adhesion and proliferation. This may facilitate better soft tissue integration between the dental implant and the surrounding bone [93].

As an alternative to more conventional materials like titanium, there has been an increase in interest in using zirconia in dental implants in recent years. The high strength and fracture toughness of zirconia make it suitable for use in long-term load-bearing dental restorations, such as dental implants. Also, zirconia has excellent biocompatibility and is corrosion-resistant, reducing the risk of implant failure [94]. Due to its many advantages, including its outstanding fracture toughness, high tensile strength, and hardness, zirconium nanoparticles have been employed in various syntheses. Five distinct isotopes of zirconium may exist naturally; out of those five, 90Zr is the most prevalent by a large margin (nearly 51.4%) [95].

In terms of esthetics, zirconia's translucency allows it to mimic the appearance of natural teeth, making it a desirable material for patients concerned about their dental restorations' appearance. This has led to increasing demand for zirconia-based dental implants, particularly in visible areas of the mouth. Despite the promising properties of zirconia, further research is necessary to fully understand the long-term performance and safety of zirconia-based dental implants. Studies have reported favourable outcomes concerning mechanical properties and biocompatibility, but further investigation is needed to establish the long-term clinical performance and safety of zirconia implants [96].

To satisfy patients' expectations for aesthetic restorations that seem natural, several different types of metal-free ceramics have been produced [97]. Zirconia has been utilized effectively as a dental biomaterial due to its biocompatibility and high mechanical qualities [98].

The dependability of ceramics in general and zirconium-based biomaterials, primarily used in biomedical and dental applications, is now the subject of extensive research. Several advancements have been concentrating on using ZTA or ATZ ceramic composites made of zirconia and alumina. These advanced composites often benefit from zirconium's transformation densification properties and are less susceptible to degrading in biological matrices at low temperatures [99].

In conclusion, using zirconium nanoparticles in dental implants offers numerous potential benefits over traditional dental implant materials. Its strength, toughness, biocompatibility, and aesthetic properties make it a promising option for dental restorations. However, further research is necessary to establish the long-term performance and safety of zirconia-based dental implants and to determine their optimal clinical use [100].

## 6 Applications of zirconium nanoparticles in dental implants

The hardness, flexibility, and fracture toughness of the denture base are significantly increased because of zirconium oxide's excellent dispersion characteristics, reduced aggregation possibility, and organic polymer biocompatibility. Dentists utilize ZrO_2_ as a filler in dental nanocomposites to improve the materials' mechanical properties, radio capacity, and appearance [101]. Researchers looked into the tri-biological behaviour of heat-cured PMMA modified with 7-wt per cent Nano-zirconium oxide. The heat-cured PMMA denture base's hardness levels, flexural strength, and fracture toughness were all dramatically increased by adding zirconium oxide nanoparticles. Nano zirconium reportedly increased the transverse strength of a repaired denture base and improved the physical qualities of denture bases during the building phase [102]. Though some disadvantages and risky factors arise from this process, research still gives high reliability on this phenomenon [103]. Press-sintering the bioactive zirconia composite layer onto the zirconia substrate resulted in success. Their findings demonstrated that due to excellent mechanical qualities and the growing interaction between the implant and living tissue, zirconia composites had a high potential to be used as a biomaterial in dental implants with a layer of bioactive zirconia composite [104]. Table 2 shows the zirconia nano textured from recent researchers.

**Table 2:** Zirconia Nano texturized surfaces: recent research [105].

Treatment on Zirconia	Methods	Study analyses	Observations	Refs
Solid-state laser	Subtractive	Bone molecular expression, in vitro and in vivo morphology and roughness studies, and osseointegration	The improved surface approach produces better outcomes in vitro and stronger forces in osseointegration tests.	[106]
Calcium phosphate nano-coating	Additive	Roughness measurements in vivo, bone-implant contact	Similar outcomes are seen with nano-coating and micro surfaces. However, High osseointegration level, when compared to control surfaces	[107]
Selective infiltration etched	Subtractive	Roughness, bone-implant contact in vivo	high levels of bone-implant contact	[108]
Nd: Yag Laser ablation + Ag/Au particles deposition	Subtractive/Additive	Morphology, atomic composition, surface functionalization, and particle adhesion in vitro	Surface functionalization using Ag and Au particles. High level of surface particle adhesion	[109]
Anodization	Subtractive	In vitro, molecular expression, morphology, roughness, and biocompatibility	Compared to control surfaces, there are significantly more bone mineralization variables	[[110], [111], [112]]
Coating Sol-gel derived TiO2	Additive	Biocompatibility in vitro	encouraging blood coagulation	[113]
Femtosecond Laser	Subtractive	Roughness, Morphology, and In vivo	Cell adhesion is substantially influenced by nano topography.	[114]
Self-assembly nanoislands	Subtractive	morphology, roughness, and biocompatibility in vitro	bone mineralization is quite high in nanosurfaces.	[115]
Hydrothermal treatment	Subtractive	Physicochemical testing	encourage surface nanotexturization	[116]

## 7 Present challenges and future opportunities

### 7.1 Present challenges

Although zirconia implants are becoming increasingly prevalent in clinical practice, they are still not a frequent treatment because there are insufficient mechanical and scientific trials [117]. Given the concern over possible nanoparticle toxicity, the assertions that nano-ZrO_2_ additions may have antioxidant and anticarcinogenic capabilities appear questionable [118]. Zirconium oxide nanoparticles have been demonstrated to damage human T cells' DNA significantly, induce apoptosis, and reduce cell growth in human mesothelioma and rodent fibroblast cell lines [[119], [120], [121]].

Zirconia has also been found to induce oxidative stress in cells, which leads to cell death. Studies have demonstrated that these NPs can stop the cell cycle, break through a number of physiological barriers, and have negative impacts [122]. Another noticeable point is due to complex surface changes, the clinical use of zirconia dental implants is constrained. Also, weak tissue interaction makes osseointegration difficult for implants with smooth surfaces [123].

Zirconium nanoparticles are currently being developed for dental implants, so more research is needed to determine how practical they will be in the clinic. Further research is required to compare their effectiveness to other materials frequently used in dental implants and find the ideal usage circumstances [124]. Zirconium nanoparticle production is still in development, and there is no set standard for doing so. The nanoparticles' characteristics and functionality may change, compromising their efficacy and safety [125].

### 7.2 Future opportunities

Zirconia (ZrO_2_), an alternative to conventional titanium-based implants for oral rehabilitation with higher aesthetic, biological, optical, and mechanical qualities, is an oxide version of zirconium, a grey-white, glossy, strong transition metal [126]. Zirconia is also quite intriguing due to its potential for osseointegration and other exceptional qualities, like a white hue and translucency that resemble genuine teeth [127]. Similar to Ti, it is radiopaque and visible under radiography. Bacterial colonization surrounding ZrO_2_ seems to be less than Ti. Compared to metallic implants (such as titanium), which are considered inert in the human body, this ceramic demonstrates negligible ion diffusion. In osseointegration, a biomaterial's surface topography and material composition are crucial [128]. One of the critical components of an implant's surface is its ability to impact osseointegration and the healing of wounds at the implant's site. Many physical and chemical alterations of surfaces have been developed to facilitate osseous healing. According to specific research, zirconia has been demonstrated to be more biocompatible than titanium, which produces corrosion products at the implant site [129]. High-strength zirconia ceramics have garnered much attention as new dental implant materials, even though titanium implants have been the subject of numerous research compared to zirconia in the past few decades [130]. Zirconia has advanced as a better dental implant material because of its biocompatibility, mechanical attributes, and tooth-like hue. Colour consistency, Using ZrO_2_ in the production of dentures has several advantages, including high strength and low thermal and electrical conductivity [131].

Zirconium nanoparticles also improved the transverse strength of a reconstructed denture base during fabrication. The investigation results indicated that a three-point bending test demonstrated that auto-polymerized resins treated with 2–5% zirconium oxide provided repairs with the highest transverse strength. According to researchers, Nano modified zirconium oxide particles in resin matrices have numerous uses, including detachable prosthodontics [132]. Also, zirconia's biocompatibility has been investigated in vivo, and when bone-implanting zirconia samples, no unfavourable reactions were noted [133]. With 100% survival and success rates for zirconia implants six months after surgery, clinical studies indicated little bone loss of roughly 2.1 mm after a year of follow-up [134].

Evidence suggests that zirconium nanoparticles can increase bone formation, reduce inflammation, have antimicrobial activity, and enhance cell adhesion and proliferation. Overall, cells react favourably to zirconium nanoparticles found in dental implants [135]. Morphological and biological tests have revealed a moderately low level of toxicity. Despite using various materials and testing techniques, no cytotoxic effects or local or systemic adverse reactions related to Zr were found [136].

## 8 Conclusion

As zirconium NPs have excellent biocompatibility and osseointegration attributes, it plays a significant role in dental implants. Zirconium nanoparticles are widely used in this discipline to enhance or resolve issues in many other fields. Zirconium nanoparticles hold promise for improving dental implants' functionality and durability, and aesthetic qualities. This review paper can increase scientific understanding of dental implants by examining recent research on zirconium NPs. The existing data can be combined, research gaps can be found, and future study options can be suggested. The use of zirconium nanoparticles in dental implants has the potential to expand the clinical practice, identify areas for development, and promote interdisciplinary cooperation. This can assist dental professionals in making decisions about using zirconium nanoparticles in dental implant procedures supported by the available data. This report examines the usage of zirconium nanoparticles in dental implants and potential improvement areas. This may assist in direct future investigations and possibly result in the creation of dental implant products and techniques that are more efficient. Much more plant species will be exploited and reported towards the simple and rapid green synthesis of metal oxide nanoparticles in the coming era. To completely comprehend their potential advantages and risks, more study is necessary. Also, there is still much room for research in areas like therapeutic dentistry and improving restorative dentistry, which have a lot of potential for using zirconium nanoparticles.

### Author contribution statement

All authors listed have significantly contributed to the development and the writing of this article.

### Data availability statement

Data will be made available on request.

### Additional information

No additional information is available for this paper.

## Declaration of competing interest

The authors declare that they have no known competing financial interests or personal relationships that could have appeared to influence the work reported in this paper.
